# Association Between Promoter Polymorphisms in CD46 and CD59 in Kidney Donors and Transplant Outcome

**DOI:** 10.3389/fimmu.2018.00972

**Published:** 2018-05-14

**Authors:** Laura A. Michielsen, Arjan D. van Zuilen, Tineke Kardol-Hoefnagel, Marianne C. Verhaar, Henny G. Otten

**Affiliations:** ^1^Department of Nephrology and Hypertension, University Medical Center Utrecht, Utrecht University, Utrecht, Netherlands; ^2^Laboratory of Translational Immunology, University Medical Center Utrecht, Utrecht University, Utrecht, Netherlands

**Keywords:** complement regulatory proteins, promoter regions, genetic, kidney donor, graft survival, acute rejection

## Abstract

Complement regulating proteins, including CD46, CD55, and CD59, protect cells against self-damage. Because of their expression on the donor endothelium, they are hypothesized to be involved in accommodation. Polymorphisms in their promoter regions may affect their expression. The aim of this study was to investigate if donor polymorphisms in complement regulating proteins influence kidney transplant outcomes. We included 306 kidney transplantations between 2005 and 2010. Five polymorphisms in the promoters of CD46, CD55, and CD59 were genotyped. A CD59 promoter polymorphism (rs147788946) in donors was associated with a lower 1-year rejection-free survival [adjusted hazard ratio (aHR) 2.18, 95% CI 1.12–4.24] and a trend toward impaired 5-year graft survival (*p* = 0.08). Patients receiving a kidney with at least one G allele for the CD46 promoter polymorphism rs2796267 (A/G) showed a lower rejection-free survival, though this became borderline significant after adjustment for potential confounders (aHR 1.87, 95% CI 0.96–3.65). A second CD46 promoter polymorphism (rs2796268, A/G), was also associated with a lower freedom from acute rejection in the presence of at least one G allele (aHR 1.95, 95% CI 1.03–3.68). Finally, the combined presence of both favorable genotypes of rs2796267 and rs147788946 had an additional protective effect both on acute rejection (*p* = 0.006) and graft survival (*p* = 0.03). These findings could help to identify patients who could benefit from intensified immunosuppressive therapy or novel complement inhibitory therapeutics.

## Introduction

Current immunologic matching of donor and recipient and risk stratification in kidney transplantation is largely based on AB0 blood group compatibility, HLA typing and the presence of donor-specific HLA antibodies, but does not include the potency of effector mechanisms such as the complement system (1–3). In kidney transplantation, complement activation can be involved in the pathogenesis of ischemia-reperfusion injury (IRI), cellular and antibody-mediated rejection, and posttransplant occurrence of certain native kidney diseases. Damage to the renal allograft is mainly mediated through the anaphylatoxins C3a and C5a, the opsonin C3b, and the membrane attack complex, resulting in pore formation in the cell membrane and subsequent cell lysis (4). To protect cells against complement-mediated damage, most nucleated cells express membrane bound complement regulatory proteins including membrane cofactor protein (CD46), decay accelerating factor (CD55), and protectin (CD59) (5). CD46 is a cofactor for factor I and thereby facilitates degradation of the complement proteins C3b and C4b. Downstream amplification of the complement cascade is further inhibited by CD55 through the inhibition of C3 convertase. Finally, CD59 interferes with the formation of the membrane attack complex by blocking the binding of C9 to C5b-C8 (5). Animal and human-biopsy studies suggest that complement regulation by these proteins on the donor endothelium is crucial in accomplishing accommodation, inducing resistance against antibody-mediated complement-dependent cell lysis, and subsequent long-term allograft function (6–10). A higher CD46 expression in renal tubules following treatment for acute T cell-mediated rejection was found to be associated with lower serum creatinine values and improved graft survival (7). Moreover, another study reported that a diffuse positive CD55 staining in the peritubulair capillaries of for cause renal allograft biopsies correlated with a smaller increase in serum creatinine and a better graft survival compared to biopsies with a negative CD55 staining (8). Finally, large-animal studies demonstrated that rejected renal grafts show lower CD59 expression compared to accommodated grafts (9, 10). The importance of these complement regulators is further illustrated by their role in the pathogenesis of atypical hemolytic uremic syndrome (aHUS) and C3 glomerulopathy, both complement dysregulation disorders (11).

Multiple studies on complement polymorphisms in kidney transplantation, including in C3, C4, and mannose-binding lectin, have been performed, but could not provide an indisputable association between these polymorphisms and acute rejection nor graft survival (12). We hypothesize that polymorphisms in genes encoding for membrane bound complement regulatory proteins in kidney donors may have a greater influence on acute rejection and graft survival because of their pivotal role in accommodation. Promoter polymorphisms can affect binding affinity of transcription factors and thereby protein expression levels (13). The genes encoding for CD46 and CD55 are located on chromosome 1 and for CD59 on chromosome 11 (14). Within the CD46 promoter region, the GG haplotype of the single-nucleotide polymorphisms (SNPs) rs2796267 and rs2796268 is associated with a lower transcriptional activity compared to the AA haplotype (15). rs2796268 is located within the consensus binding sequence of the transcription factor CBF-1/RBP-Jk (15). Therefore, donor kidneys with at least one G allele for these SNPs may show a lower CD46 expression upon their endothelium compared to kidneys with a homozygous A genotype. Both G alleles are also part of larger aHUS risk haplotype (16). A 21-bp deletion in the promoter region of CD55 (rs150046210) is associated with a lower transcriptional activity and CD55 expression levels and with more severe influenza infections and allergic respiratory diseases (17, 18). Finally, an adenine insertion in the promoter of CD59 (rs147788946) in lung donors is associated with the incidence of bronchiolitis obliterans syndrome (BOS) following lung transplantation (19). The aim of this study was to investigate whether donor polymorphisms in the promoters of CD46, CD55, and CD59 affect kidney transplant outcomes.

## Patients and Methods

### Patients and Study Design

Between January 2005 and December 2010, 357 transplantations were performed in the UMC Utrecht. 51 transplantations were excluded because no donor DNA was available (*n* = 38) or donor DNA was degraded and not suitable for genotyping anymore (*n* = 13), leaving 306 transplantations for this analysis. Clinical data were obtained from hospital records and the Dutch Organ Transplant Registry for which all patients provided written informed consent. Posttransplant follow-up data were available for all patients for at least 5 years. All patients provided consent for use of leftover sera samples. The primary outcomes in this study were 5-year death-censored graft survival and 1-year freedom from acute rejection. For acute rejection, we decided to look at 1-year freedom from acute rejection because the incidence of acute rejection is the highest within the first year and rejection after the first year is often related with nonadherence or overaggressive immunosuppressive tapering (20). Rejection was defined as biopsy proven acute borderline rejection or acute rejection. All biopsies were performed on indication and reviewed by an experienced nephropathologist according to the Banff classification valid at that time. The study protocol including usage of donor DNA and patient sera was approved by the Biobank Research Ethics Committee of the UMC Utrecht (TC Bio 13-633) and performed in accordance with the Declaration of Helsinki.

### Anti-HLA Antibodies

In all transplantations, the T-cell complement-dependent cytotoxicity crossmatch with both current and peak sera was negative. In addition, pretransplant anti-HLA antibodies were previously determined with the LABScreen panel reactive antigen and single antigen class I and II (OneLambda, CA, USA) for all transplantations between 2005 and September 2008 (21). For transplantations performed after September 2008, sera were retrospectively tested for the presence of anti-HLA antibodies with the LifeScreen Deluxe and Lifecodes single antigen beads class I and II (Immucor, GA, USA) in case of a positive screening. Based on a comparative study between both HLA antibody assays have a similar ability to detect anti-HLA antibodies at a mean fluorescence intensity cutoff of 4,000 (22), we decided to apply this cutoff. Donor-specific anti-HLA antibodies (DSA) were assigned for HLA-A/-B/-C/-DR/-DQ by comparing bead specificities with the donor HLA type on split level.

### Donor DNA Isolation and Genotyping

Donor DNA was extracted from peripheral blood mononuclear cells or splenocytes at the time of transplantation for HLA typing purposes by using the MagnaPure Compact system according to the manufacturer’s instructions (Roche diagnostics, Basel, Switzerland) and stored at 4°C. Because all donor DNA samples were stored with a donor code that was not traceable to an identifiable person, permission to use the leftover DNA samples for study purposes was granted by the Biobank Research Ethics Committee of the UMC Utrecht. Genomic DNA was amplified through polymerase chain reaction (PCR) by using specific primer-pairs for the promoters of CD46, CD55, and CD59 (Table S1 in Supplementary Material). Following enzymatic purification, the PCR products were sequenced by using sequence-primers and fluorescent capillary electrophoresis (3730 DNA analyzer, Applied Biosystems, Waltham, MA, USA). Sequence data were analyzed with SeqScape^®^ version 2.7 (Applied Biosystems).

### Statistical Analyses

All data were analyzed with SAS Enterprise Guide 7.1 (SAS Institute Inc., Cary, NC, USA) and R 3.2.2. Survival analyses were performed by constructing Kaplan–Meier curves and tested for significance with the log-rank test. No correction for multiple testing was performed because we selected the investigated polymorphisms beforehand based on literature and frequency within the general population instead of random testing of all identified polymorphisms within the CD46, CD55, and CD59 promoter regions (23). To adjust for potential confounders, cox multiple regression was performed. Included in the adjusted analysis were panel reactive antibody (PRA), donor type, retransplantation, and induction therapy. Results are reported as hazard ratios (HRs) with 95% confidence interval and *p*-values. A *p*-value of <0.05 was considered to be statistically significant.

## Results

### Patient and Donor Characteristics

Five different polymorphisms in the promoters of CD46, CD55, and CD59 that are frequently present within the general population were sequenced. The observed genotype frequencies within our donor population are summarized in Table 1 and are comparable to the frequencies that have been reported by the 1000 genomes project (14). We will refer to the two different CD46 SNPs as A (rs2796267) and B (rs2796268). Additional donor and recipient characteristics are summarized in Table 2. Fifty-eight patients suffered from at least one episode of biopsy proven acute rejection within the first year. These episodes were classified as borderline rejection (12%), acute cellular rejection (66%), acute antibody-mediated rejection (5%), or combined antibody-mediated and cellular rejection (17%). The overall death-censored 5-year graft survival rate was 84%, 48 grafts failed during follow-up. Thirty-one patients died with a functioning graft within 5 years posttransplantation.

**Table 1:** Overview of studied polymorphisms.

Complement protein	Polymorphism—rs number	Alleles	Genotype frequencies within cohort
CD46	2796267 (A)	A/G	A/A: 31% G/G: 17% A/G: 52%
	2796268 (B)	A/G	A/A: 31% G/G 17% A/G: 52%

CD55	150046210 (A)	−/TAGTTACTTCCCCTCCTTCCC	+/+: 49% −/−: 43% +/−: 9%
	28371583 (B)	A/G	A/A: 54% G/G: 8% A/G: 38%

CD59	147788946	−/A	−/−: 71% A/−: 29%

**Table 2:** Baseline characteristics.

	Cohort (*n* = 306)
Recipient age (years)	49.6 ± 13.8
Recipient sex, male	172 (56%)
Donor age (years)	51.5 ± 13.2
Donor sex, male	137 (45%)
Donor type	
Living	136 (44%)
DBD	85 (28%)
DCD	85 (28%)
First transplant	257 (84%)
Highest PRA > 5%	57 (19%)
Pretransplant DSA^b^	33 (11%)
HLA-A, -B, -DR mismatches (no.)	
0–1	72 (24%)
2–4	189 (62%)
5–6	45 (15%)
Cold ischemia time (hours)^c^	16.5 ± 6.8
Delayed graft function^a^	77 (25%)
Baseline immunosuppression	
Tacrolimus	299 (98%)
Cyclosporine A	2 (1%)
Mycophenolate mofetil	277 (91%)
Azathioprine	2 (1%)
Prednisone	303 (99%)
Sirolimus	20 (7%)
Induction therapy^d^	54 (18%)

*Data are depicted as number and percentage or mean ± SD*.

*DBD, donation after brain death; DCD, donation after circulatory death; DSA, donor-specific anti-HLA antibodies; PRA, panel reactive antigen*.

*^a^Defined as the need for dialysis indicated by poor kidney function within the first week after transplantation*.

*^b^Pretransplant DSA status could not be determined for five patients*.

*^c^Cold ischemia time for deceased donors only*.

*^d^Induction therapy with anti-interleukin 2 receptor monoclonal antibody*.

### CD46 and CD59 Promoter Polymorphisms Are Associated With Acute Rejection

**Figure 1:**
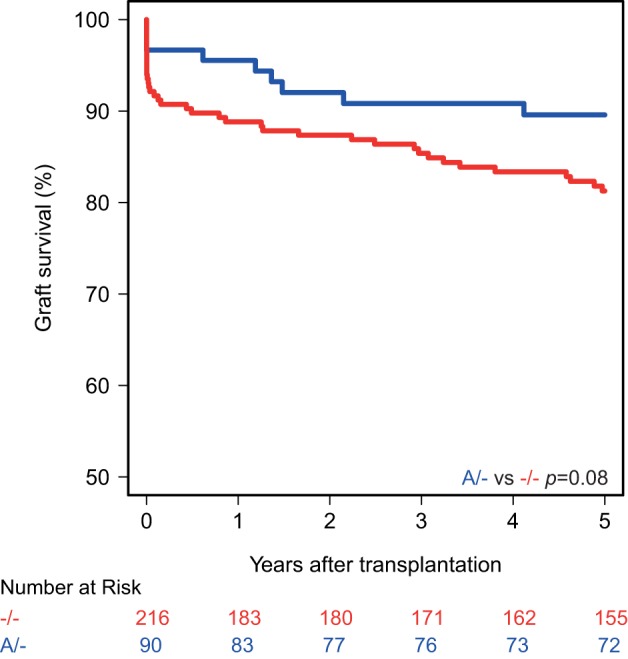
Death-censored graft survival curve according to donor CD59 promoter genotype. Kidneys with the −/− single-nucleotide polymorphism configuration tend to have an impaired 5-year death-censored graft survival (*p* = 0.08).

**Figure 2:**
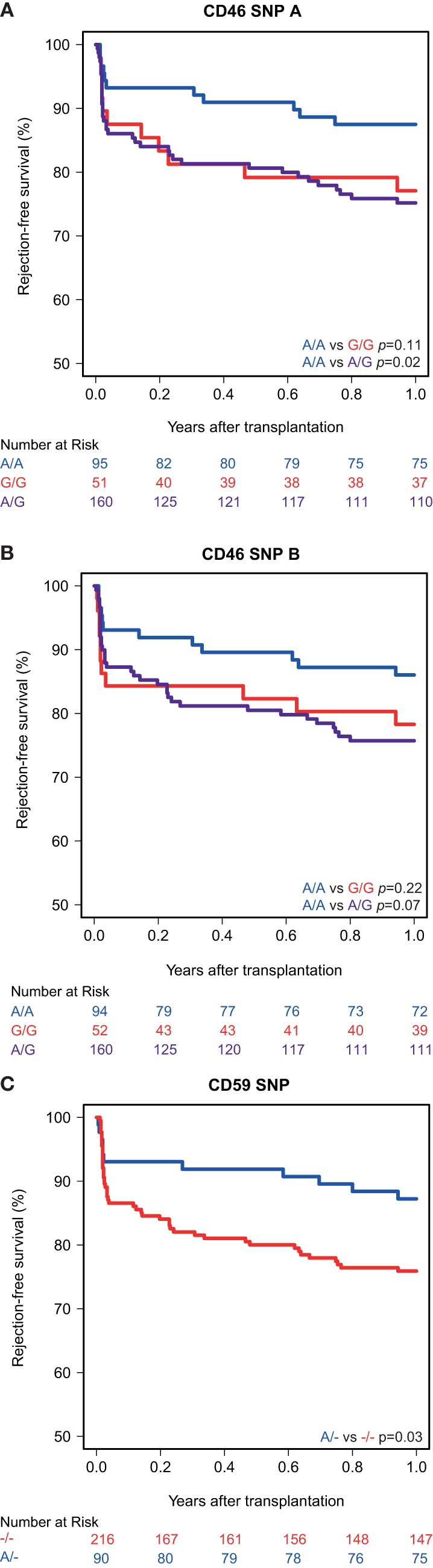
Rejection-free survival according to donor CD46 and CD59 genotypes. **(A)** Rejection-free survival according to CD46 SNP A (rs2796267): A/A vs. G/G (*p* = 0.11), A/A vs. A/G (*p* = 0.02), A/A vs. G/R (*p* = 0.02). **(B)** Rejection-free survival according to CD46 SNP B (rs2796268): A/A vs. G/G (*p* = 0.22), A/A vs. A/G (*p* = 0.07), A/A vs. G/R (*p* = 0.07). **(C)** Rejection-free survival according to CD59 SNP (rs147788946): −/− vs. A/− (*p* = 0.03).

Kaplan–Meier survival analyses showed no associations between the polymorphisms in the promoters of the genes encoding for CD46 and CD55 and 5-year death-censored graft survival. For the CD59 promoter polymorphism, the survival curve hints at an impaired survival of kidneys with a SNP configuration without an adenine insertion (−/−), although this is not significant (*p* = 0.08; Figure 1). Regarding freedom from acute rejection, differences were observed for the CD46 and CD59 promoter polymorphisms, but not for CD55 (data not shown). Patients receiving a kidney with at least one G allele for the CD46 SNP A showed a significantly lower freedom from acute rejection (*p* = 0.02; Figure 2A). The other CD46 SNP (B), showed a trend towards a lower freedom from acute rejection in the presence of at least one G allele (*p* = 0.07; Figure 2B). Finally, the −/− configuration of the CD59 SNP in kidney donors correlated also with an impaired rejection-free survival (*p* = 0.03; Figure 2C). The observed differences for all three SNPs occured already within the first weeks posttransplantation. Types of rejection stratified for donor SNP genotype are summarized in Table S2 in Supplementary Material.

Recipient and transplant characteristics did not significantly differ among CD59 and CD46 SNP B donor genotype (Tables S3 and S4 in Supplementary Material). For CD46 SNP A, patients receiving a kidney with at least one G allele tended to be more often retransplant patients and subsequently also had more often a PRA above 5% and induction therapy with an IL-2 receptor antagonist (Table S4 in Supplementary Material). After adjustment for donor type, PRA, retransplantation and induction therapy in a cox multiple regression model, there was still a trend toward a lower rejection-free survival (HR 1.82; 0.93–3.55) in patients receiving a kidney from a donor with at least one G allele for CD46 SNP A (Table 3). The adjusted analyses, including the same covariates as for CD46 SNP A, identified CD46 SNP B and CD59 SNP as significant risk markers for acute rejection within the first year (HR 1.95 and 2.18).

**Table 3:** Hazard ratios (HRs) for acute rejection and graft failure.

Promoter polymorphism	Rs number	Genotype	Acute rejection^a^		Graft failure^b^	
			Unadjusted	Adjusted^c^	Unadjusted	Adjusted^c^
CD46 SNP A	2796267	G/R vs. A/A	2.09 (1.09–4.03)	1.87 (0.96–3.65)	1.10 (0.59–2.05)	1.03 (0.55–1.95)
CD46 SNP B	2796268	G/R vs. A/A	1.80 (0.95–3.39)	1.95 (1.03–3.68)	0.88 (0.48–1.61)	0.89 (0.40–1.64)
CD59	147788946	−/− vs. A/−	2.01 (1.04–3.87)	2.18 (1.12–4.24)	1.88 (0.91–3.88)	1.88 (0.90–3.89)

*Data depicted as HRs with 95% confidence interval*.

*SNP, single-nucleotide polymorphism*.

*^a^Acute rejection within the first year*.

*^b^5-year death-censored graft failure*.

*^c^Adjusted for panel reactive antibodies, donortype, retransplantation, and induction therapy*.

Because DSA are important inducers of complement activation, we also looked at the presence of pretransplant DSA in combination with donor genotypes. Five-year graft survival was the lowest in patients with pretransplant DSA receiving a kidney with a CD59 risk genotype (64%, overall *p* = 0.02; Figure S1 in Supplementary Material). Moreover, rejection-free survival in patients with DSA was lower in patients receiving a kidney with a CD59 risk genotype compared to a protective genotype (*p* = 0.03; Figure S2 in Supplementary Material). For CD46 SNP A and B, no difference in rejection-free survival in patients with DSA was observed between the risk and protective genotypes, though in patients without DSA rejection-free survival was lower for the risk genotypes.

### Protective Complotype Yields Additional Preservative Effects

**Figure 3:**
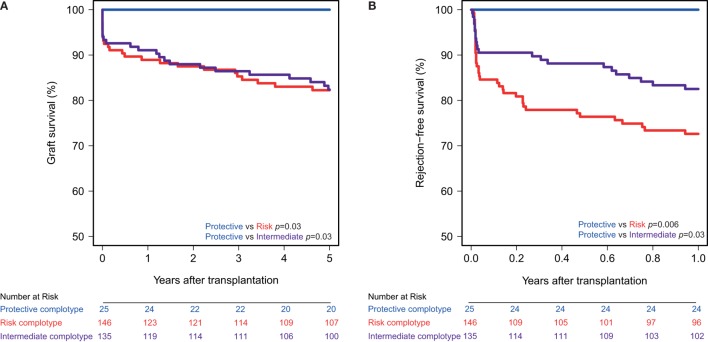
Graft and rejection-free survival according to donor complotype. Protective complotype (CD46 SNP A: A/A and CD59 SNP: A/−), risk complotype (CD46 SNP A: G/R and CD59 SNP: −/−), and intermediate complotype (CD46 SNP A: G/R and CD59 SNP: A/− or CD46 SNP A: A/A and CD59 SNP: −/−). **(A)** 5-year death-censored graft survival according to donor complotype: protective vs. risk complotype (*p* = 0.03) and protective vs. intermediate complotype (*p* = 0.03). **(B)** 1-year rejection-free survival according to donor complotype: protective vs. risk complotype (*p* = 0.006), protective vs. intermediate complotype (*p* = 0.03), and intermediate vs. risk complotype (*p* = 0.05).

The combined presence of multiple complement polymorphisms, a complotype, may yield additional information. Therefore, we compared patients receiving a kidney with both protective variants of CD46 SNP A and CD59 SNP alongside kidneys with both risk variants and kidneys with one protective and one risk variant, the intermediate group. Recipient and transplant characteristics of all groups are summarized in Table 4, failure causes in Table S5 in Supplementary Material. Remarkably, none of the patients receiving a kidney from a donor with both protective genotypes suffered from acute rejection within the first year nor failed within 5 years (Figure 3). Kidneys with a risk or intermediate complotype showed an identical lower 5-year graft survival compared to kidneys with a protective complotype (82%, *p* = 0.03). Regarding 1-year rejection-free survival, a dose-dependent effect was observed, with a lower rejection-free survival in kidneys with the risk complotype (73%) compared to kidneys with an intermediate complotype (83%, *p* = 0.05).

**Table 4:** Baseline characteristics according to donor complotype.

	Protective complotype	Risk complotype	Intermediate risk complotype
	
	*N* = 25	*N* = 146	*N* = 135
Recipient age (years)	50.2 ± 12.1	50.8 ± 12.0	48.3 ± 14.9
Recipient sex, male	17 (68%)	83 (57%)	72 (53%)
Donor age (years)	52.8 ± 14.8	50.6 ± 13.9	52.2 ± 12.2
Donor sex, male	11 (44%)	68 (47%)	58 (43%)
Donor type			
Living	7 (28%)	62 (43%)	67 (50%)
DBD	6 (24%)	44 (30%)	35 (26%)
DCD	12 (48%)	40 (27%)	33 (24%)
First transplant	22 (88%)	117 (80%)	118 (87%)
Highest PRA > 5%	3 (12%)	30 (21%)	24 (19%)
Pretransplant DSA^a^	4 (16%)	18 (13%)	11 (8%)
HLA-A, -B, -DR mismatches (no.)			
0–1	6 (24%)	33 (23%)	33 (24%)
2–4	15 (60%)	91 (62%)	83 (62%)
5–6	4 (16%)	22 (15%)	19 (14%)
Cold ischemia time (hours)^c^	16.4 ± 9.2	17.0 ± 6.8	16.3 ± 6.2
Delayed graft function^b^	11 (44%)	35 (24%)	31 (23%)
Induction therapy^d^	1 (4%)	27 (19%)	26 (19%)

*Baseline characteristics stratified for protective complotype (CD46 SNP A: A/A and CD59 SNP: −/−), risk complotype (CD46 SNP A: G/R and CD59 SNP: A/−), or intermediate risk complotype (CD46 SNP A: A/A and CD59 SNP: A/− or CD46 SNP A: G/R and CD59 SNP: −/−). Data are depicted as number and percentage or mean ± SD*.

*DBD, donation after brain death; DCD, donation after circulatory death; DSA, donor-specific anti-HLA antibodies; PRA, panel reactive antibodies; SNP, single-nucleotide polymorphism*.

*^a^Data on pretransplant DSA status was missing for four patients receiving a kidney with a risk complotype and one patient with a donor with an intermediate complotype*.

*^b^Defined as the need for dialysis indicated by poor kidney function within the first week after transplantation*.

*^c^Cold ischemia time for deceased donors only*.

*^d^Induction therapy with anti-interleukin 2 receptor monoclonal antibody*.

## Discussion

This study demonstrated that two promoter polymorphisms in CD46 and one in CD59 in kidney donors correlate with a lower freedom from acute rejection within the first year posttransplantation. The survival analyses hint at a lower 5-year graft survival in patients receiving a kidney with the −/− configuration of the CD59 SNP, although this was not significant. The combined presence of both protective genotypes of CD46 SNP A and CD59 SNP has an additional preservative effect on freedom from acute rejection and 5-year graft survival. There was no association between the CD55 promoter polymorphisms and rejection-free nor graft survival.

Mechanisms by which CD46 can protect against both cellular and antibody-mediated rejection include decreased production of complement C3a and C5a and inhibition of further complement activation and amplification. Locally produced C3a and C5a can bind to antigen-presenting cells (APCs) and T cells, thereby inhibiting T-cell apoptosis and enhancing T-cell proliferation and the production of co-stimulatory molecules and cytokines by APCs (4, 24–27). CD46 also acts as a direct co-stimulatory molecule for T cells, driving them towards the anti-inflammatory type 1 regulatory phenotype (28, 29). The observed early effect of both CD46 SNPs on rejection-free survival, in the absence of DSA, may be the result of enhanced T-cell activation and proliferation upon IRI induced complement activation (30).

Donor CD59 protects the allograft against rejection by hindering the formation of the membrane attack complex. Membrane attack complex formation on the donor endothelium can results in lysis, whereas sublytic levels induce endothelial cell activation and altered proliferation (31–33), augment alloreactive T-cell activation (34), and promote a prothrombotic phenotype (35–37). In addition, CD59 inhibits activation and proliferation of CD4+ and CD8+ T cells (38). Because of the limited numbers and involvement of complement in both cellular and antibody-mediated rejection, we decided to pool the rejection subsets. The majority of the additional rejection episodes in the CD59 risk genotype group were classified as cellular rejection. In a substantial part of these biopsies, vascular rejection was observed which is classically seen as a type of cellular rejection. However, in the presence of DSA this could also indicate antibody-mediate rejection. Indeed, for CD59 it seems like both graft failure and acute rejection within the first days posttransplantation are most prevalent in the presence of both pretransplant DSA and the donor CD59 risk genotype (39). Pretransplant DSA have been associated with an increased risk of early failure (40). This effect may be attributed to increased HLA antigen expression on the donor kidney within the first days due to IRI (40). Moreover, IRI also activates the complement cascade directly (30) and this may further contribute to the observed early effect of the complement polymorphisms.

To the best of our knowledge, this is the first study that assessed the impact of promoter polymorphisms in genes encoding for complement regulatory proteins in kidney donors on transplant outcomes. Park et al. studied the CD46 SNP A in kidney transplant recipients and reported an inverse association between this polymorphism and acute and late-onset acute rejection in kidney transplant recipients (41). However, the pathological relevance of this polymorphism in recipients in terms of acute rejection is less clear since accommodation is primarily mediated by local complement regulatory proteins expressed on the donor organ (42). In our study, the −/− configuration of the CD59 SNP in kidney donors yielded a disadvantageous association with acute rejection. Previously, in a cohort of 137 lung transplantations, of whom 14 were diagnosed with BOS, a reverse association between the CD59 promoter polymorphism in lung donors and BOS was observed (19). A potential explanation for the opposite effect of this SNP in kidney and lung transplantation could apply to the timing of the effect and the fact that acute and chronic rejection (including BOS) are two distinct entities (43–45). Chronic rejection is a much more multifactorial process requiring multiple hits, resulting in gradual parenchymal fibrosis and obliterative vasculopathy and progressive graft dysfunction (43, 46), whereas acute rejection is identified by cellular and humoral attack resulting in rapid graft damage when untreated (43, 47). In our kidney transplant cohort, the observed effect of the CD59 promoter SNP occurred in the first weeks posttransplantation without an effect thereafter. Therefore, we hypothesize that the CD59 promoter with the −/− configuration might be more inducible through vigorous inflammation such as IRI, whereas the other CD59 variant might be more strongly transcribed in steady state. Finally, the effect of the CD59 promoter SNP may be tissue specific. It was shown that not all cell types are equally responsive to stimuli such as phorbol myristate acetate, an NF-κB activator (48), when it comes to CD59 upregulation (49). Further studies should reveal the molecular effect of the studied CD59 promoter polymorphism.

This discovery study was set up to explore the potential associations between promoter polymorphisms in the genes encoding for complement regulatory proteins and kidney transplant outcomes. A limitation of this study is the relatively small sample size for a SNP association study and lack of replication cohort. The sample size has likely also attributed to differences in patient characteristics (retransplantation, PRA, and induction therapy) when stratifying for CD46 SNP A donor genotypes, since we would not expect an association between these factors and a donor polymorphism. Retransplantation and PRA are general markers of immunization and can include both cellular and humoral immunization (1). We tried to overcome this potential bias by adjusting for these factors in cox multiple regression. Validation of our results in a larger, adequately powered, replication cohort is required to strengthen our findings. The incidence of rejection within the first and 5-year graft survival observed in our population are in line with numbers reported by others (2, 50). Death-censored graft survival among the 51 patients transplanted without donor DNA who were excluded from further analysis, seemed to be slightly better compared to patients with available donor DNA (93%, *p* = 0.08). However, when taking death with functioning graft into account, no significant differences in 5-year graft survival were observed between patients with available donor DNA (74%) and without donor DNA (65%, *p* = 0.25).

It has been postulated that the combined presence of multiple polymorphisms in complement genes, a complotype, could have an even greater impact on complement activity (51–53). For example, in age-related macular degeneration, a complement dysregulation disorder, a combination of a SNP in factor H and two in factor B shows the strongest association with disease status and complement activity *in vitro* (52). Therefore, we constructed a complotype combining the CD46 SNP A and CD59 SNP. The combined presence of both protective genotypes was associated with additional beneficial outcome compared to kidneys with only a single protective variant. Moreover, none of the kidneys with both protective variants showed any signs of acute rejection within the first year nor failed during follow-up. Because of the limited number of patients receiving a kidney with a protective complotype and absence of events in this group, we could not adjust for baseline differences by cox multiple regression. Donors with a protective complotype were more often donation after cardiac death donors and less often living donors. On the other hand, patients receiving a kidney with a protective complotype also had a lower PRA and received less often induction therapy. A potential bias in both directions can therefore not be ruled out.

In conclusion, the presented data suggests that donor polymorphisms in the promoters of CD46 and CD59 affect kidney transplant outcomes. This study opens new perspectives on the role of complement regulation in preventing acute rejection and graft failure and could add valuable information to already known risk indicators of unfavorable outcomes following kidney transplantation. We hypothesize that kidneys with a risk complotype are less capable of protecting themselves against recipient-induced complement attack. Therefore, these patients may benefit from complement-targeted therapeutics like eculizumab and complement C1-inhibitor or one of the newly developed inhibitors that are currently being investigated (54). Alongside this information could also help to determine which patients could benefit from more intensified regular immunosuppressive treatment and more frequent check ups.

## Ethics Statement

Clinical data were obtained from hospital records and the Dutch Organ Transplant Registry for which all patients provided written informed consent. The study protocol was approved by the Biobank Research Ethics Committee of the UMC Utrecht (TC Bio 13-633) and performed in accordance with the Declaration of Helsinki.

## Author Contributions

TK-H and LM performed the research; AZ, LM, HO, and MV participated in data analysis; AZ, LM, and HO participated in research design; AZ, LM, HO, and MV wrote the paper. All authors provided final approval of the version to be published.

## Conflict of Interest Statement

AZ has received a travel grant and speakers fee from Astellas Pharma and is on the Dutch Novartis Transplant Advisory Board. LM has received a travel grant from Astellas Pharma. All other authors have no conflict of interest to disclose.

## Supplementary Material

Figure S1 Death-censored graft survival according to donor CD59 promoter genotype and pretransplant DSA status. 5-year graft survival was comparable between patients with a protective (A/−) genotype kidney without DSA (90%) and with DSA (91%). For the CD59 risk genotype (−/−), graft survival was 83% in patients without DSA and 64% in patients with DSA (overall *p* = 0.02).

Figure S2 Rejection-free survival according to donor CD46 and CD59 genotypes in combination with pretransplant DSA status. **(A,B)** For CD46 single-nucleotide polymorphism (SNP) A and SNP B, rejection-free survival did not differ between the protective (A/A) and risk (A/G or G/G) variant in patients with pretransplant DSA. Whereas, rejection-free survival was lower in patients without DSA receiving a kidney with a risk genotype of CD46 SNP A (*p* = 0.02) or CD46 SNP B (*p* = 0.06). **(C)** For CD59, rejection-free survival in patients with DSA was markedly lower for the CD59 risk genotype (−/−; *p* = 0.03). In patients without DSA, rejection-free survival was 87% in patients with a protective genotype (A/−) and 80% in patients with a risk genotype (*p* = 0.16).

## Abbreviations

BOS, bronchiolitis obliterans syndrome; CD46, membrane cofactor protein; CD55, decay accelerating factor; CD59, protectin; DSA, donor-specific anti-HLA antibodies; HR, hazard ratio; PCR, polymerase chain reaction; PRA, panel reactive antibodies; SNP, single-nucleotide polymorphism.

## References

[1] Otten HG Joosten I Allebes WA van der Meer A Hilbrands LB Baas M The PROCARE consortium: toward an improved allocation strategy for kidney allografts. Transpl Immunol (2014) 31(4):184–90. 10.1016/j.trim.2014.09.008 25258025

[2] Matas AJ Smith JM Skeans MA Thompson B Gustafson SK Schnitzler MA OPTN/SRTR 2012 annual data report: kidney. Am J Transplant (2014) 14(Suppl 1):11–44. 10.1111/ajt.12579 24373166

[3] Glorie K Haase-Kromwijk B van de Klundert J Wagelmans A Weimar W. Allocation and matching in kidney exchange programs. Transpl Int (2014) 27:333–43. 10.1111/tri.12202 24112284

[4] Sacks SH Zhou W. The role of complement in the early immune response to transplantation. Nat Rev Immunol (2012) 12:431–42. 10.1038/nri3225 22627861

[5] Zipfel PF Skerka C. Complement regulators and inhibitory proteins. Nat Rev Immunol (2009) 9:729–40. 10.1038/nri2620 19730437

[6] Lynch RJ Platt JL. Accommodation in renal transplantation: unanswered questions. Curr Opin Organ Transplant (2010) 15:481–5. 10.1097/MOT.0b013e32833b9c25 20613524 PMC3085890

[7] Yamanaka K Kakuta Y Miyagawa S Nakazawa S Kato T Abe T Depression of complement regulatory factors in rat and human renal grafts is associated with the progress of acute T-cell mediated rejection. PLoS One (2016) 11:e0148881. 10.1371/journal.pone.0148881 26928779 PMC4771804

[8] Brodsky SV Nadasdy GM Pelletier R Satoskar A Birmingham DJ Hadley GA Expression of the decay-accelerating factor (CD55) in renal transplants—a possible prediction marker of allograft survival. Transplantation (2009) 88:457–64. 10.1097/TP.0b013e3181b0517d 19696627

[9] Griesemer AD Okumi M Shimizu A Moran S Ishikawa Y Iorio J Upregulation of CD59: potential mechanism of accommodation in a large animal model. Transplantation (2009) 87:1308–17. 10.1097/TP.0b013e3181a19afc 19424030 PMC2772119

[10] Chen Song S Zhong S Xiang Y Li JH Guo H Wang WY Complement inhibition enables renal allograft accommodation and long-term engraftment in presensitized nonhuman primates. Am J Transplant (2011) 11:2057–66. 10.1111/j.1600-6143.2011.03646.x 21831160

[11] Barbour S Gill JS. Advances in the understanding of complement mediated glomerular disease: implications for recurrence in the transplant setting. Am J Transplant (2015) 15:312–9. 10.1111/ajt.13042 25612487

[12] Michielsen LA van Zuilen AD Muskens IS Verhaar MC Otten HG. Complement polymorphisms in kidney transplantation: critical in graft rejection? Am J Transplant (2017) 17:2000–7. 10.1111/ajt.14199 28097805

[13] de Córdoba SR Tortajada A Harris CL Morgan BP. Complement dysregulation and disease: from genes and proteins to diagnostics and drugs. Immunobiology (2012) 217:1034–46. 10.1016/j.imbio.2012.07.021 22964229

[14] 1000 Genomes Project Consortium Auton A Brooks LD Durbin RM Garrison EP Kang HM A global reference for human genetic variation. Nature (2015) 526:68–74. 10.1038/nature15393 26432245 PMC4750478

[15] Esparza-Gordillo J Goicoechea de Jorge E Buil A Carreras Berges L López-Trascasa M Sánchez-Corral P Predisposition to atypical hemolytic uremic syndrome involves the concurrence of different susceptibility alleles in the regulators of complement activation gene cluster in 1q32. Hum Mol Genet (2005) 14:703–12. 10.1093/hmg/ddi066 15661753

[16] Moore I Strain L Pappworth I Kavanagh D Barlow PN Herbert AP Association of factor H autoantibodies with deletions of CFHR1, CFHR3, CFHR4, and with mutations in CFH, CFI, CD46, and C3 in patients with atypical hemolytic uremic syndrome. Blood (2010) 115:379–87. 10.1182/blood-2009-05-221549 19861685 PMC2829859

[17] Kawai T Takeshita S Imoto Y Matsumoto Y Sakashita M Suzuki D Associations between decay-accelerating factor polymorphisms and allergic respiratory diseases. Clin Exp Allergy (2009) 39:1508–14. 10.1111/j.1365-2222.2009.03316.x 19681921

[18] Lin T-Y Brass AL. Host genetic determinants of influenza pathogenicity. Curr Opin Virol (2013) 3:531–6. 10.1016/j.coviro.2013.07.005 23933004 PMC4127448

[19] Budding K van de Graaf EA Kardol-Hoefnagel T Broen JCA Kwakkel-van Erp JM Oudijk EJD A promoter polymorphism in the CD59 complement regulatory protein gene in donor lungs correlates with a higher risk for chronic rejection after lung transplantation. Am J Transplant (2016) 16:987–98. 10.1111/ajt.13497 26517734

[20] Sellares J de Freitas DG Mengel M Reeve J Einecke G Sis B Understanding the causes of kidney transplant failure: the dominant role of antibody-mediated rejection and nonadherence. Am J Transplant (2011) 12:388–99. 10.1111/j.1600-6143.2011.03840.x 22081892

[21] Otten HG Verhaar MC Borst HPE Hené RJ van Zuilen AD. Pretransplant donor-specific HLA class-I and -II antibodies are associated with an increased risk for kidney graft failure. Am J Transplant (2012) 12:1618–23. 10.1111/j.1600-6143.2011.03985.x 22404993

[22] Clerkin KJ See SB Farr MA Restaino SW Serban G Latif F Comparative assessment of anti-HLA antibodies using two commercially available luminex-based assays. Transplant Direct (2017) 3:e218. 10.1097/TXD.0000000000000734 29184907 PMC5682763

[23] Streiner DL Norman GR. Correction for multiple testing: is there a resolution? Chest (2011) 140:16–8. 10.1378/chest.11-0523 21729890

[24] Li K Patel H Farrar CA Hargreaves REG Sacks SH Zhou W. Complement activation regulates the capacity of proximal tubular epithelial cell to stimulate alloreactive T cell response. J Am Soc Nephrol (2004) 15:2414–22. 10.1097/01.ASN.0000135974.06478.7B 15339990

[25] Peng Q Li K Anderson K Farrar CA Lu B Smith RAG Local production and activation of complement up-regulates the allostimulatory function of dendritic cells through C3a-C3aR interaction. Blood (2008) 111:2452–61. 10.1182/blood-2007-06-095018 18056835

[26] Strainic MG Liu J Huang D An F Lalli PN Muqim N Locally produced complement fragments C5a and C3a provide both costimulatory and survival signals to naive CD4+ T cells. Immunity (2008) 28:425–35. 10.1016/j.immuni.2008.02.001 18328742 PMC2646383

[27] Raedler H Yang M Lalli PN Medof ME Heeger PS. Primed CD8(+) T-cell responses to allogeneic endothelial cells are controlled by local complement activation. Am J Transplant (2009) 9:1784–95. 10.1111/j.1600-6143.2009.02723.x 19563342

[28] Ni Choileain S Astier AL. CD46 processing: a means of expression. Immunobiology (2012) 217:169–75. 10.1016/j.imbio.2011.06.003 21742405 PMC4363545

[29] Charron L Doctrinal A Ni Choileain S Astier AL. Monocyte: T-cell interaction regulates human T-cell activation through a CD28/CD46 crosstalk. Immunol Cell Biol (2015) 93:796–803. 10.1038/icb.2015.42 25787182 PMC4519525

[30] Farrar CA Asgari E Schwaeble WJ Sacks SH. Which pathways trigger the role of complement in ischaemia/reperfusion injury? Front Immunol (2012) 3:341. 10.3389/fimmu.2012.00341 23181062 PMC3500775

[31] Tegla CA Cudrici C Patel S Trippe R Rus V Niculescu F Membrane attack by complement: the assembly and biology of terminal complement complexes. Immunol Res (2011) 51:45–60. 10.1007/s12026-011-8239-5 21850539 PMC3732183

[32] Bayly-Jones C Bubeck D Dunstone MA. The mystery behind membrane insertion: a review of the complement membrane attack complex. Philos Trans R Soc Lond B Biol Sci (2017) 372:20160221. 10.1098/rstb.2016.0221 28630159 PMC5483522

[33] Morgan BP Boyd C Bubeck D. Molecular cell biology of complement membrane attack. Semin Cell Dev Biol (2017) 72:124–32. 10.1016/j.semcdb.2017.06.009 28647534

[34] Jane-wit D Manes TD Yi T Qin L Clark P Kirkiles-Smith NC Alloantibody and complement promote T cell-mediated cardiac allograft vasculopathy through noncanonical nuclear factor-B signaling in endothelial cells. Circulation (2013) 128:2504–16. 10.1161/CIRCULATIONAHA.113.002972 24045046 PMC3885874

[35] Hamilton KK Hattori R Esmon CT Sims PJ. Complement proteins C5b-9 induce vesiculation of the endothelial plasma membrane and expose catalytic surface for assembly of the prothrombinase enzyme complex. J Biol Chem (1990) 265:3809–14. 2105954

[36] Sims PJ Wiedmer T Induction of cellular procoagulant activity by the membrane attack complex of complement. Semin Cell Biol (1995) 6:275–82. 10.1006/scel.1995.0037 8562920

[37] Karpman D Ståhl A-L Arvidsson I Johansson K Loos S Tati R Complement interactions with blood cells, endothelial cells and microvesicles in thrombotic and inflammatory conditions. Adv Exp Med Biol (2015) 865:19–42. 10.1007/978-3-319-18603-0_2 26306441

[38] Xie X-H Gao M-H Zhang B Wang M-J Wang J. Post-transcriptional CD59 gene silencing by siRNAs induces enhanced human T lymphocyte response to tumor cell lysate-loaded DCs. Cell Immunol (2012) 274:1–11. 10.1016/j.cellimm.2012.02.013 22480874

[39] Rodríguez Cubillo B Pérez Flores I Calvo N Pascual A Cortés JA Moreno MA Antibody-mediated acute vascular rejection of kidney allografts: fifteen-year follow-up. Transplant Proc (2016) 48:2917–9. 10.1016/j.transproceed.2016.09.015 27932107

[40] Kamburova EG Wisse BW Joosten I Allebes WA van der Meer A Hilbrands LB Differential effects of donor-specific HLA antibodies in living- versus deceased-donor transplantation. Am J Transplant (2018) 1–11. 10.1111/ajt.14709 PMC6175247 29464832

[41] Park MS Kim SK Lee TW Lee SH Moon JY Ihm CG Promoter polymorphism in the CD46 complement regulatory protein gene is associated with acute renal allograft rejection. Transplant Proc (2016) 48:809–12. 10.1016/j.transproceed.2015.12.126 27234742

[42] Touzot M Obada EN Beaudreuil S Francois H Durrbach A Complement modulation in solid-organ transplantation. Transplant Rev (Orlando) (2014) 28:119–25. 10.1016/j.trre.2014.03.001 24996770

[43] Chalasani G Li Q Konieczny BT Smith-Diggs L Wrobel B Dai Z The allograft defines the type of rejection (acute versus chronic) in the face of an established effector immune response. J Immunol (2004) 172:7813–20. 10.4049/jimmunol.172.12.7813 15187165

[44] Kaul AMK Goparaju S Dvorina N Iida S Keslar KS la Motte de CA Acute and chronic rejection: compartmentalization and kinetics of counterbalancing signals in cardiac transplants. Am J Transplant (2015) 15:333–45. 10.1111/ajt.13014 25582188 PMC4304877

[45] Verleden GM Raghu G Meyer KC Glanville AR Corris P. A new classification system for chronic lung allograft dysfunction. J Heart Lung Transplant (2014) 33:127–33. 10.1016/j.healun.2014.01.316 24374027

[46] Aguilar PR Michelson AP Isakow W. Obliterative bronchiolitis. Transplantation (2016) 100:272–83. 10.1097/TP.0000000000000892 26335918

[47] Bröcker V Mengel M. Histopathological diagnosis of acute and chronic rejection in pediatric kidney transplantation. Pediatr Nephrol (2014) 29:1939–49. 10.1007/s00467-013-2640-3 24141526

[48] Chang M-S Chen B-C Yu M-T Sheu J-R Chen T-F Lin C-H. Phorbol 12-myristate 13-acetate upregulates cyclooxygenase-2 expression in human pulmonary epithelial cells via Ras, Raf-1, ERK, and NF-kappaB, but not p38 MAPK, pathways. Cell Signal (2005) 17:299–310. 10.1016/j.cellsig.2004.07.008 15567061

[49] Holguin MH Martin CB Eggett T Parker CJ Analysis of the gene that encodes the complement regulatory protein, membrane inhibitor of reactive lysis (CD59). Identification of an alternatively spliced exon and characterization of the transcriptional regulatory regions of the promoter. J Immunol (1996) 157:1659–68. 8759753

[50] Golshayan D Wójtowicz A Bibert S Pyndiah N Manuel O Binet I Polymorphisms in the lectin pathway of complement activation influence the incidence of acute rejection and graft outcome after kidney transplantation. Kidney Int (2016) 89:927–38. 10.1016/j.kint.2015.11.025 26924055

[51] Harris CL Heurich M Rodríguez de Córdoba S Morgan BP. The complotype: dictating risk for inflammation and infection. Trends Immunol (2012) 33:513–21. 10.1016/j.it.2012.06.001 22749446 PMC3460238

[52] Paun CC Lechanteur YTE Groenewoud JMM Altay L Schick T DAHA MR A novel complotype combination associates with age-related macular degeneration and high complement activation levels in vivo. Sci Rep (2016) 6:26568. 10.1038/srep26568 27241480 PMC4886525

[53] Lay E Nutland S Smith JE Hiles I Smith RAG Seilly DJ Complotype affects the extent of down-regulation by factor I of the C3b feedback cycle in vitro. Clin Exp Immunol (2015) 181:314–22. 10.1111/cei.12437 25124117 PMC4516447

[54] Jager NM Poppelaars F Daha MR Seelen MA. Complement in renal transplantation: the road to translation. Mol Immunol (2017) 89:22–35. 10.1016/j.molimm.2017.05.014 28558950

[55] Michielsen L van Zuilen A Kardol-Hoefnagel T Verhaar M Otten H Two promoter polymorphisms in the genes encoding for complement regulating proteins CD46 and CD59 in kidney donors are associated with biopsy proven acute rejection. Am J Transplant (2017) 17(Suppl 3). 10.1111/ajt.14306

