# Supplementary figures and images for: Association Between Promoter Polymorphisms in CD46 and CD59 in Kidney Donors and Transplant Outcome
Source: Front Immunol. 2018 May 14;9:972. doi: 10.3389/fimmu.2018.00972 (PMC5960667; doi:10.3389/fimmu.2018.00972)

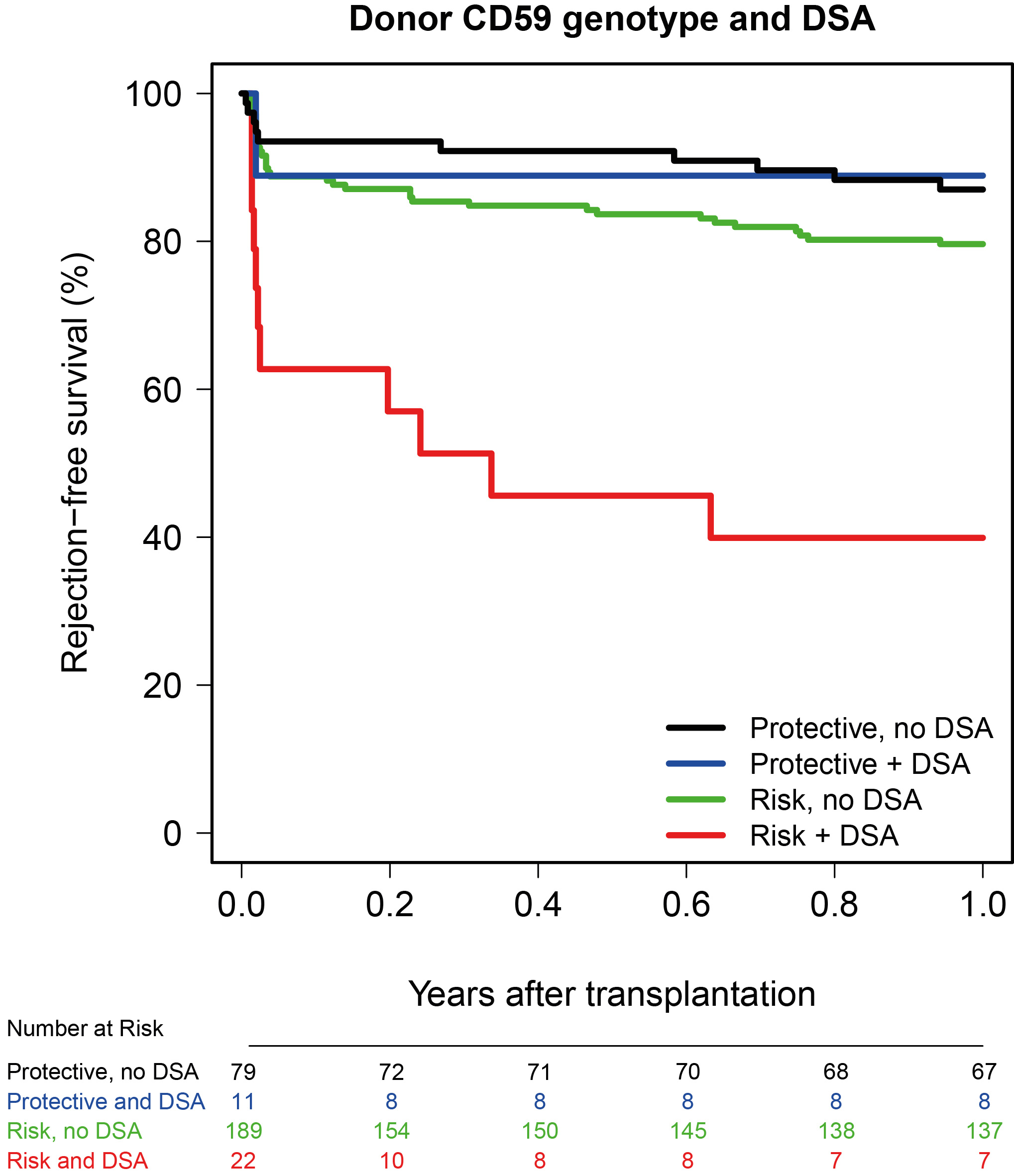

Supplement: Figure S1 — Death-censored graft survival according to donor CD59 promoter genotype and pretransplant DSA status. 5-year graft survival was comparable between patients with a protective (A/−) genotype kidney without DSA (90%) and with DSA (91%). For the CD59 risk genotype (−/−), graft survival was 83% in patients without DSA and 64% in patients with DSA (overall p = 0.02). [file image_1.jpeg]

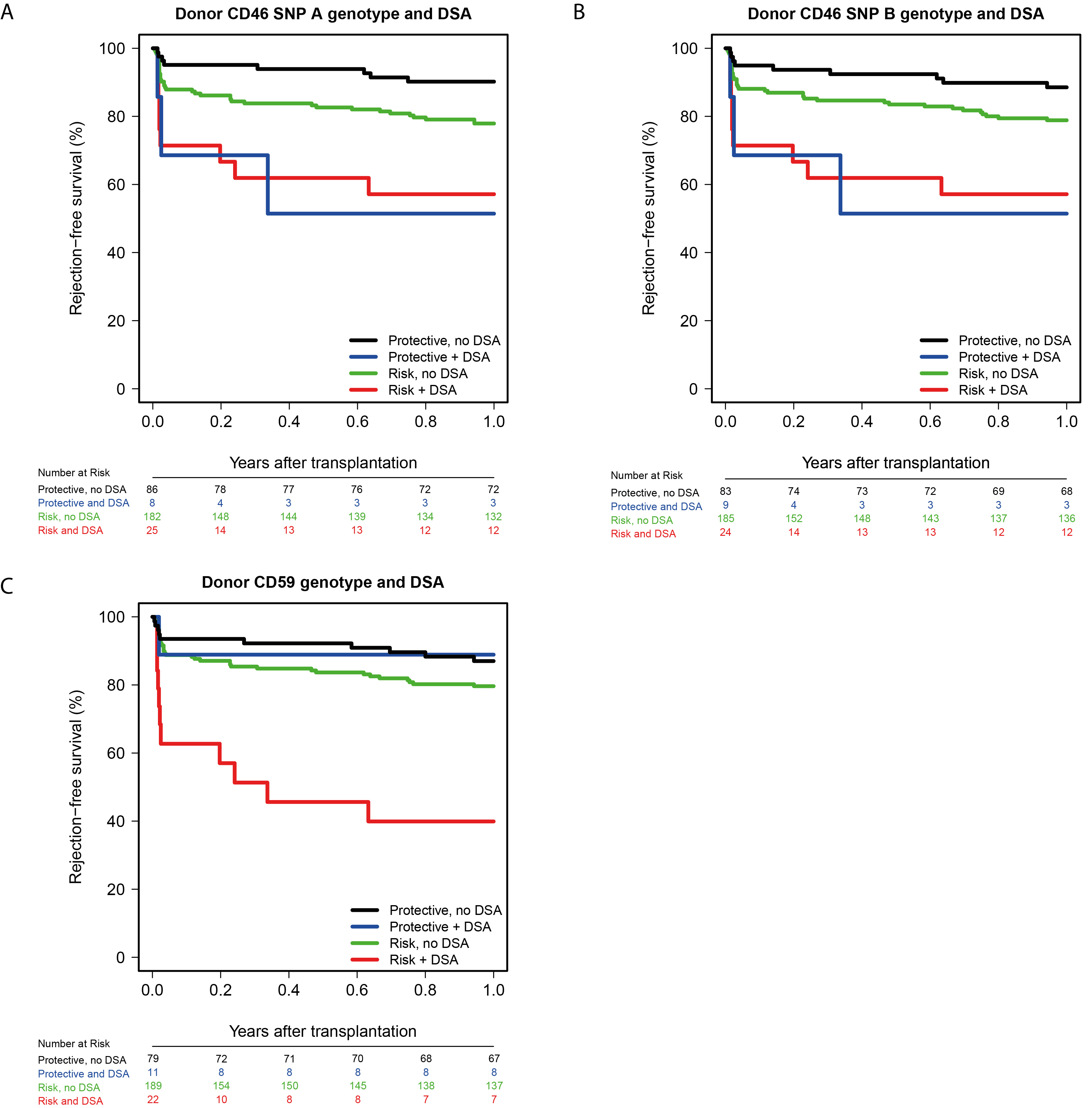

Supplement: Figure S2 — Rejection-free survival according to donor CD46 and CD59 genotypes in combination with pretransplant DSA status. (A,B) For CD46 single-nucleotide polymorphism (SNP) A and SNP B, rejection-free survival did not differ between the protective (A/A) and risk (A/G or G/G) variant in patients with pretransplant DSA. Whereas, rejection-free survival was lower in patients without DSA receiving a kidney with a risk genotype of CD46 SNP A (p = 0.02) or CD46 SNP B (p = 0.06). (C) For CD59, rejection-free survival in patients with DSA was markedly lower for the CD59 risk genotype (−/−; p = 0.03). In patients without DSA, rejection-free survival was 87% in patients with a protective genotype (A/−) and 80% in patients with a risk genotype (p = 0.16). [file image_2.jpeg]
