# Supplementary material for: Association Between Promoter Polymorphisms in CD46 and CD59 in Kidney Donors and Transplant Outcome
Source: Front Immunol. 2018 May 14;9:972. doi: 10.3389/fimmu.2018.00972 (PMC5960667; doi:10.3389/fimmu.2018.00972)
Supplement: Supplementary file 3 [file table_1.docx]

**Supplementary table 1: primer sequences**

| **Complement protein** | **Primer** | **Direction** | **Sequence** |
| --- | --- | --- | --- |
| CD46 | PCR | Forward | 5'-TGGCACTTAGGACACCCTT-3' |
|  |  | Reverse | 5'- CACCATGGCCGCCAGA-3' |
|  | Sequence | Forward | 5'-GCTTAATAAATTAGATTCGGAAGGG-3' |
| CD55 | PCR | Forward | 5'-ATTGTTATCCCACCCCACAC-3' |
|  |  | Reverse | 5'-GCACAACAGCACCAGCAG-3' |
|  | Sequence | Reverse | 5'-ACACCCTCTGGGACTCACTCT-3' |
| CD59 | PCR | Forward | 5'-TGTAAAACGACGGCCAGTGACCCAATGAGCACCTTCAAA-3' |
|  |  | Reverse | 5'-CAGGAAACAGCTATGACCGGCTGTGGCTGATGGCTATT-3' |
|  | Sequence | Forward | 5'-GTAAAACGACGGCCAG-3' |

Primer sequences used for genomic amplification by polymerase chain reaction (PCR). PCR products were sequence in a single direction by using specific sequence primers.
