# Supplementary material for: Association Between Promoter Polymorphisms in CD46 and CD59 in Kidney Donors and Transplant Outcome
Source: Front Immunol. 2018 May 14;9:972. doi: 10.3389/fimmu.2018.00972 (PMC5960667; doi:10.3389/fimmu.2018.00972)
Supplement: Supplementary file 4 [file table_2.docx]

**Supplementary table 2: Overview of the types of acute rejection stratified for donor genotype**

|  | **CD59** | | **CD46 SNP A** | | | **CD46 SNP B** | | |
| --- | --- | --- | --- | --- | --- | --- | --- | --- |
|  | -/- | A/- | A/A | A/G | G/G | A/A | A/G | G/G |
| Number of patients | 216 | 90 | 95 | 51 | 160 | 94 | 52 | 160 |
| Rejection | 48 (22%) | 11 (12%) | 11 (12%) | 11 (22%) | 37 (23%) | 12 (13%) | 11 (21%) | 36 (23%) |
| Rejection type: | | |  |  |  |  |  |  |
| Cellular | 31 (14%) | 8 (9%) | 5 (5%) | 8 (16%) | 26 (16%) | 8 (9%) | 7 (13%) | 24 (15%) |
| Antibody-mediated | 2 (1%) | 1 (1%) | 1 (1%) | 0 | 2 (1%) | 1 (1%) | 0 | 2 (1%) |
| Mixed | 8 (4%) | 2 (2%) | 2 (2%) | 1 (2%) | 7 (4%) | 1 (1%) | 3 (6%) | 6 (4%) |
| Borderline | 7 (3%) | 0 | 3 (3%) | 2 (4%) | 2 (1%) | 2 (2%) | 1 (2%) | 4 (3%) |

Only first rejection episodes within the first year are summarized in this table, data are depicted as number and percentage.
