# Supplementary material for: Association Between Promoter Polymorphisms in CD46 and CD59 in Kidney Donors and Transplant Outcome
Source: Front Immunol. 2018 May 14;9:972. doi: 10.3389/fimmu.2018.00972 (PMC5960667; doi:10.3389/fimmu.2018.00972)
Supplement: Supplementary file 5 [file table_3.docx]

**Supplementary Table 3: Cohort characteristics according to donor CD59 genotypes**

|  | **Entire cohort** | **CD59 -/-** | **CD59 A/-** |
| --- | --- | --- | --- |
|  | N = 306 | N = 216 | N = 90 |
| Recipient age (years) | 49.6 ± 13.8 | 50.3 ± 12.8 | 47.9 ± 14.5 |
| Recipient sex, male | 172 (56%) | 120 (56%) | 52 (58%) |
| Donor age (years) | 51.5 ± 13.2 | 51.6 ± 13.4 | 51.9 ± 12.9 |
| Donor sex, male | 137 (45%) | 99 (46%) | 38 (42%) |
| Donor type  Living  DBD  DCD | 136 (44%)  85 (28%)  85 (28%) | 93 (43%)  65 (30%)  58 (27%) | 43 (48%)  20 (22%)  27 (30%) |
| First transplant | 257 (84%) | 183 (85%) | 74 (83%) |
| Highest PRA >5% | 57 (19%) | 37 (17%) | 20 (22%) |
| Pretransplant DSA^^^ | 33 (11%) | 22 (10%) | 11 (12%) |
| HLA-A, -B, -DR mismatches (no.)  0-1  2-4  5-6 | 72 (24%)  189 (62%)  45 (15%) | 49 (23%)  137 (63%)  30 (14%) | 23 (26%)  52 (58%)  15 (17%) |
| Cold ischemia time (hours)* | 16.5 ± 6.8 | 16.5 ± 6.7 | 16.5 ± 7.1 |
| Delayed graft function^#^ | 77 (25%) | 52 (24%) | 25 (28%) |
| Baseline immunosuppression  Tacrolimus  Cyclosporine A  Mycophenolate mofetil  Azathioprine  Prednisone  Sirolimus | 299 (98%)  2 (1%)  277 (91%)  2 (1%)  303 (99%)  20 (7%) | 211 (98%)  0  200 (97%)  2 (1%)  213 (99%)  8 (4%) | 87 (98%)  2 (2%)  77 (86%)  0  90 (100%)  12 (13%) |
| Induction therapy^§^ | 54 (18%) | 35 (16%) | 19 (21%) |

Data are depicted as number and percentage or mean ± standard deviation.

Abbreviations: DBD, donation after brain death; DCD, donation after circulatory death; PRA, panel reactive antibody.

* Cold ischemia time for deceased donors

^^^ Pretransplant DSA status could not be determined for 5 patients

^#^ Defined as the need for dialysis indicated by poor kidney function within the first week after transplantation.

^§^ Induction therapy with anti-Interleukin 2 receptor monoclonal antibody
