# Supplementary material for: Association Between Promoter Polymorphisms in CD46 and CD59 in Kidney Donors and Transplant Outcome
Source: Front Immunol. 2018 May 14;9:972. doi: 10.3389/fimmu.2018.00972 (PMC5960667; doi:10.3389/fimmu.2018.00972)
Supplement: Supplementary file 6 [file table_4.docx]

**Supplementary table 4: Cohort characteristics according to donor CD46 genotypes**

|  | **CD46 SNP A** | | | **CD46 SNP B** | | |
| --- | --- | --- | --- | --- | --- | --- |
| Genotype | A/A | G/G | A/G | A/A | G/G | A/G |
|  | N=95 | N=51 | N=160 | N=94 | N=52 | N=160 |
| Recipient age (years) | 49.6 ± 13.9 | 54.2 ± 12.0* | 48.2 ± 13.2 | 49.4 ± 13.9 | 52.7 ± 12.8 | 48.8 ± 13.2 |
| Recipient sex, male | 54 (57%) | 26 (51%) | 92 (58%) | 54 (57%) | 30 (58%) | 88 (55%) |
| Donor age (years) | 52.8 ± 12.9 | 51.8 ± 12.0 | 50.6 ± 13.8 | 51.5 ± 14.5 | 52.6 ± 12.9 | 51.1 ± 12.6 |
| Donor sex, male | 42 (44%) | 22 (43%) | 73 (46%) | 41 (44%) | 19 (37%) | 77 (48%) |
| Donor type  Living  DBD  DCD | 38 (40%)  27 (28%)  30 (32%) | 22 (43%)  15 (29%)  14 (28%) | 76 (48%)  43 (27%)  41 (26%) | 35 (37%)  27 (29%)  32 (34%) | 24 (46%)  16 (31%)  12 (23%) | 77 (48%)  42 (26%)  41 (26%) |
| First transplant | 88 (93%) | 43 (84%) | 126 (79%)* | 83 (88%) | 45 (87%) | 129 (81%) |
| Highest PRA >5% | 10 (11%) | 9 (18%) | 38 (24%)* | 17 (18%) | 10 (19%) | 30 (19%) |
| Pretransplant DSA^^^ | 8 (9%) | 7 (15%) | 18 (12%) | 9 (10%) | 8 (16%) | 16 (10%) |
| HLA-A, -B, -DR mismatches (no.)  0-1  2-4  5-6 | 22 (23%)  61 (64%)  12 (13%) | *  16 (31%)  22 (43%)  13 (26%) | 34 (21%)  106 (66%)  20 (13%) | 22 (23%)  63 (67%)  9 (10%) | 13 (25%)  31 (60%)  8 (15%) | 37 (23%)  95 (59%)  28 (18%) |
| Cold ischemia time (hr)^±^ | 16.3 ± 6.3 | 16.9 ± 5.2 | 16.6 ± 7.7 | 15.9 ± 7.4 | 17.3 ± 5.3 | 16.7 ± 6.9 |
| Delayed graft function^#^ | 28 (29%) | 12 (24%) | 37 (23%) | 28 (30%) | 13 (25%) | 36 (23%) |
| Baseline immunosuppression  Tacrolimus  Cyclosporine A  Mycophenolate mofetil  Azathioprine  Prednisone  Sirolimus | 93 (98%)  0  87 (92%)  0  93 (98%)  5 (5%) | 49 (96%)  1 (2%)  47 (92%)  0  51 (100%)  4 (8%) | 157 (98%)  1 (1%)  143 (89%)  2 (1%)  159 (99%)  11 (7%) | 92 (98%)  0  82 (87%)  0  92 (98%)  8 (9%) | 50 (96%)  1  46 (88%)  0  52 (100%)  5 (10%) | 157 (98%)  1 (1%)  149 (93%)  2 (1%)  159 (99%)  7 (2%) |
| Induction therapy^§^ | 9 (10%) | 12 (26%)* | 32 (20%)* | 13 (14%) | 12 (23%) | 29 (18%) |

Data are depicted as number and percentage or mean ± standard deviation.

Abbreviations: DBD, donation after brain death; DCD, donation after circulatory death; DSA, donor-specific anti-HLA antibodies, PRA, panel reactive antibody.

* Statistically significant compared to CD46 SNP A A/A genotype

^^^ Pretransplant DSA status could not be determined for 5 patients.

^±^ Cold ischemia time for deceased donors only

^#^ Defined as the need for dialysis indicated by poor kidney function within the first week after transplantation.

^§^ Induction therapy with anti-Interleukin 2 receptor monoclonal antibody
