# Supplementary material for: Association Between Promoter Polymorphisms in CD46 and CD59 in Kidney Donors and Transplant Outcome
Source: Front Immunol. 2018 May 14;9:972. doi: 10.3389/fimmu.2018.00972 (PMC5960667; doi:10.3389/fimmu.2018.00972)
Supplement: Supplementary file 7 [file table_5.docx]

**Supplementary table 5: Failure causes according to complotype**

|  | **Intermediate complotype** | **Risk complotype** |
| --- | --- | --- |
|  | N=23 | N=25 |
| Rejection | 12 (52%) | 14 (56%) |
| Thrombosis | 6 (26%) | 5 (20%) |
| Primary non-function | 3 (13%) | 6 (24%) |
| Other | 2 (9%)* | 0 |

Data are depicted as number and percentage. No failures were observed in the protective complotype group.

* Renal cell carcinoma in allograft, infection
